# Oral health profile of postbariatric surgery individuals: A case series

**DOI:** 10.1002/cre2.411

**Published:** 2021-03-05

**Authors:** Negin Taghat, Karin Mossberg, Peter Lingström, Sofia Björkman, Anna Lehrkinder, Malin Werling, Anna‐Lena Östberg

**Affiliations:** ^1^ Department of Behavioural and Community Dentistry, Institute of Odontology, The Sahlgrenska Academy University of Gothenburg Gothenburg Sweden; ^2^ Institute of Medicine, The Sahlgrenska Academy University of Gothenburg Gothenburg Sweden; ^3^ Department of Cariology, Institute of Odontology, The Sahlgrenska Academy University of Gothenburg Gothenburg Sweden; ^4^ Department of Gastrosurgical Research and Education, Sahlgrenska Academy University of Gothenburg Gothenburg Sweden; ^5^ Department of Gastroenterology and Hepatology, Unit of Clinical Nutrition and The Regional Obesity Center Sahlgrenska University Hospital Gothenburg Sweden

**Keywords:** bariatric surgery, case series, oral health

## Abstract

**Objectives:**

To describe the oral health profile of individuals who had undergone gastric bypass surgery (GBP) or sleeve gastrectomy (SG) to generate hypotheses for further studies.

**Material and Methods:**

Fourteen individuals treated with GBP or SG surgery ≥ 2 years ago and with observed and/or perceived oral problems were recruited to a case series. The documentation included clinical and radiographic examinations, biomedical sampling, and self‐reported diet and questionnaires. The results are presented descriptively.

**Results:** The age range was 31 to 66 years and all had a BMI > 25 (range 25.4–44.7). Only four participants were fully dentate. Eleven out of 14 individuals exhibited severe decay. A majority had poor oral hygiene and high bacterial counts. The flow rates of unstimulated saliva were extremely low and hyposalivation was present in ten of the fourteen cases. Most perceived several oral health problems, such as chewing difficulty and tooth hypersensitivity.

**Conclusions:**

Individuals who had undergone GBP or SG surgery had poor clinically diagnosed oral health and perceived oral health problems. Longitudinal studies are needed to monitor the patients' oral health, from before bariatric surgery to long‐term postoperatively.

## 1 INTRODUCTION

Obesity is a global health issue today with worldwide rates tripling since 1975. In 2016, 650 million adults (13%) were reported as obese that is, a Body Mass Index (BMI) greater than or equal to 30 kg/m^2^ (WHO, 2020). The corresponding proportion in Sweden is 15%, according to a recent national health survey (The Public Health Agency of Sweden, 2018). Obesity is considered a risk factor for an array of chronic diseases (The Global Burden of Disease 2015 Collaborators, 2017) however, studies on the association between an obese condition and oral disease show somewhat contradictory results and causal relationships are not well established (Nascimento et al., 2016; Shivakumar, Srivastava, & Shivakumar, 2018).

Obesity is difficult to treat despite several treatment options, including reduced dietary energy intake, physical activity, pharmaceutical treatment, and surgery (Bray, Fruhbeck, Ryan, & Wilding, 2016). Bariatric surgery is the most effective treatment for morbid obesity that is a BMI≥40 kg/m^2^ or a BMI≥35 kg/m^2^ in presence of obesity‐related comorbidities f.i. diabetes or hypertension (NIH conference, 1991), and results in effective weight loss and reduced comorbidities (O'Brien et al., 2019). The number of surgeries has increased steadily in recent decades with gastric bypass surgery (GBP) and sleeve gastrectomy (SG) being the most frequently used surgical methods today (Angrisani et al., 2018). GBP involves formation of a small pouch from the upper part of the stomach and connecting it to the small intestine, enabling ingested food to pass directly into the intestinal tract. With SG, the greater part (3/4) of the stomach is removed and the rest of the stomach is formed into a narrow tube. Before qualifying for surgery, subjects need to reduce their body weight 5–10% through dietary modifications, to minimize intra‐ and postoperative complications (Anderin, Gustafsson, Heijbel, & Thorell, 2015).

Despite the many benefits of bariatric surgery, the restructuring of the gastrointestinal tract may cause complications. The short‐term complications are mainly due to the surgical procedure, while long‐term complications are the consequence of the restrictive and malabsorptive process that occurs after bariatric surgery. Known long‐term complications are i.a. abdominal pain, gastroesophageal reflux disease (GERD) and nutritional deficiencies (Schulman et al., 2017). However, the long‐term adverse effects are not fully elucidated (Höskuldsdottir et al., 2020).

The impact of bariatric surgery on oral health is sparsely studied and the findings contradictory. Some studies indicate associations between bariatric surgery and periodontal disease (Marsicano, Sales‐Peres, Ceneviva, & Sales‐Peres, 2012; Sales‐Peres et al., 2015), dental caries (Hague & Baechle, 2008; Marsicano, Grec, Belarmino, Ceneviva, & Peres, 2011; Salgado‐Peralvo et al., 2018) and tooth hypersensitivity (Netto et al., 2012), while others did not (Cardozo et al., 2014; Jaiswal et al., 2015). Biomedical characteristics and oral behaviors after surgery need for example to be explored. The studies have often focused on a single problem f.i. dental caries; however, there is no overall picture of possible oral problems facing the post‐bariatric individual. Hence, knowledge in the area is fragmented.

Taken together, the oral health problems in post‐bariatric individuals need to be thoroughly described in order to generate hypotheses for longitudinal studies in the field. Thus, the aim of the present study was to describe the oral health profile of individuals who had undergone the most common bariatric surgery procedures, gastric bypass or sleeve gastrectomy.

## 2 MATERIAL AND METHODS

A case series was designed (*A Dictionary of Epidemiology*, 2014). The inclusion criteria were having undergone GBP or SG surgery (Angrisani et al., 2018) ≥2 years earlier, together with professionally observed and/or patient‐perceived aggravated oral problems after surgery. The exclusion criteria were treatment with other bariatric surgeries than GBP or SG or individuals who had their original bariatric procedure rearranged to GBP or SG. The surgical technique used was verified by medical records. After surgery, all had been asked to adhere to standardized daily nutritional substitution adapted to age and gender according to guidelines (Laurenius, Näslund, Sandvik, Videhult, & Wirén, 2018).

The Regional Ethical Review Board in Gothenburg (reg. no. 237‐16) approved the study and all participants provided written consent.

### 2.1 Procedure and variables

Recruitment took place through a network of dental practitioners and health care professionals, such as physicians, dieticians and nurses in the western part of Sweden, who in their work identified patients with oral health problems after bariatric surgery. This selection generated a kind of convenience sample.

The participants filled out a 4‐day food record prior to the dental visit. They were instructed to eat according to their normal everyday meal patterns while keeping a detailed record during four consecutive days (three weekdays and one weekend day). Instructions were also given to express the quantities using common household measures and kitchen scales, and to provide detailed descriptions of the food items, portion sizes and type of meals. The food records were analyzed by a registered dietician (SB) using the software Dietist Net Pro® and the National Food Composition Tables version 20171215. Meal frequencies and total daily energy intake were calculated and the distribution of the nutrient content was analyzed.

At the dental visit, the participants first filled in a questionnaire. Oral health habits were represented by tooth brushing (twice a day, vs. once a day/a few times a week/seldom/never), interdental cleaning (everyday/3–5 times a week, vs. 1–2 times a week/never), frequency of dental appointments during the last 5 years (regularly/at least once a year/3–4 appointments, vs. 1–2 appointments/never), and the reason for the last dental visit (routine examination, vs. pain/other problem). Possible oral problems included tooth hypersensitivity, acid reflux episodes and vomiting episodes (never/a few times a year, vs. a few times a month/a few times a week/daily). The participants rated their chewing ability (without difficulty, vs. difficult/unable). Their Oral Health‐Related Quality of Life (OHRQoL) was assessed using the Oral Health Impact Profile (OHIP‐14) (Slade, 1997). The response options for each item were “never” (0), “hardly ever” (1), “occasionally” (2), “fairly often” (3) and “very often” (4) with a possible maximum of 56, with a higher score indicating a greater impact.

A medical history was recorded in connection with the dental examination. Height (cm) and weight (kg) wearing light clothes were registered and Body Mass Index (BMI) was calculated (kg/m^2^).

Thereafter, unstimulated salivary secretion rate was measured for 15 minutes. Stimulated saliva was collected for 5 min with the participant actively chewing a paraffin pellet and spitting continuously. The salivary buffer capacity (low/medium/high) of the stimulated saliva was determined chairside (CRT Buffer^®^). Total bacterial count, total streptococci count, streptococcus mutans (SM) and lactobacilli, all per colony‐forming unit (CFU)/ml of stimulated saliva were analyzed at the laboratory of the Department of Cariology, Institute of Odontology, University of Gothenburg, Sweden. Salivary electrolytes were analyzed at the Clinical Chemistry laboratory, Sahlgrenska University Hospital, Gothenburg, Sweden (Klingberg et al., 2007).

The clinical oral examination was then performed by one of two calibrated dentists (NT and ALÖ). The third molars were excluded from all examinations.

Dental caries was registered by visual and radiographic examination. Visual assessments of all tooth surfaces (buccal, lingual, distal, mesial, occlusal) were made according to the International Caries Assessment System II (ICDAS) (Ismail et al., 2007) with scores from 0 (sound surface) to 6 (distinct excessive cavity with visible dentine). Restorations and missing teeth were noted. The radiographic assessment was performed on four bitewing radiographs according to ICDAS, on three surfaces of the molars and premolars (distal, mesial, occlusal), and scored from 0 (no radiolucency) to 6 (radiolucency into the pulp). Detailed coding schemes are attached as Table S1. Clinical and radiographic assessments were weighed together for all surfaces recorded on radiographs. When the deviation between the clinical and the radiological assessment was maximum one step, the higher recorded value was used. When the difference was greater, the radiographs were reexamined (number of surfaces = 36). All these differences occurred when caries was diagnosed on radiographs under old fillings; that is, difficult to detect clinically.

Periodontal status was represented by (a) visible dental plaque, 0/1; (b) gingivitis, bleeding on probing, (0/1) and (c) probing pocket depths (measured from the gingival margin): healthy (<3.5 mm) or pathological (≥ 3.5 mm) (Löe, 1967; Ramfjord, 1967).

### 2.2 Statistical analysis

Data management and analyses were carried out using SPSS version 25. Results are presented with descriptive statistics including mean values, *SDs* and minimum‐maximum scores.

## 3 RESULTS

A total of 14 subjects were examined, with an age range of 31–66 years. Six participants were younger than 40 years, five were between 40 and 59 years old and three were 60 years or older. Two were males. All participants had a BMI > 25 (range 25.4–44.7) at the time of the clinical examination. They had undergone bariatric surgery 2–19 years ago (mean 13 years) (Table 1). The majority had been treated with GBP and one with SG (case 14). All reported adhering to the standardized substitution therapy recommended after bariatric surgery (Höskuldsdottir et al., 2020; Laurenius et al., 2018). Six of the participants stated regular use of prescription medication, such as painkillers and antidepressants. All had a minimum of compulsory education (9 years) and all but four were cohabiting (not in tables).

**TABLE 1:** Description of the sample

Case	Age span	Gender	Surgical technique	Years since operation	Included via	Main experienced oral problem after surgery	BMI at clinical examination after surgery
1	40–59	Female	GBP	>2 years^a^	Dietician	Fractures, brittle teeth	38.9
2	21–39	Male	GBP	10	Dietician	Tooth hypersensitivity	26.0
3	21–39	Female	GBP	9	Physician	Tooth hypersensitivity	^a^
4	21–39	Female	GBP	11	Physician	Bleeding gums	39.5
5	60–66	Female	GBP	8	Dietician	Xerostomia	40.4
6	60–66	Female	GBP	13	Dietician	Tooth decay, periodontitis	30.3
7	40–59	Female	GBP	13	Dietician	Tooth decay	44.7
8	40–59	Female	GBP	19	Dentist	Tooth decay, brittle teeth	25.9
9	40–59	Female	GBP	11	Dentist	Periodontitis	31.6
10	60–66	Female	GBP	19	Dentist	Tooth decay, brittle teeth	34.7
11	40–59	Female	GBP	9	Dentist	Tooth decay	25.4
12	21–39	Female	GBP	9	Dentist	Tooth decay, brittle teeth	32.5
13	21–39	Female	GBP	12	Nurse	Tooth decay, brittle teeth	35.4
14	21–39	Male	SG	2	Dentist	Tooth decay	27.8

^a^ Insufficient data.

The number of remaining teeth ranged between 16 and 28 (mean 24), with only four participants being fully dentate. The proportion of clinically registered dental caries of any stage, ICDAS codes 1‐6, on the total number of examined surfaces in all participants was 26% (not in tables). Two‐thirds of all lesions were categorized as initial non‐cavitated caries (ICDAS 1‐2).

Dental caries and periodontal status, for all 14 participants separately, are presented in Table 2. All participants exhibited dental caries with the number of decayed surfaces ranging between 7 and 80. Eleven of the fourteen participants showed severely decayed surfaces (ICDAS 5‐6). Two participants had nine and one participant had ten such surfaces. All participants had previous restorations (filled surfaces mean 29.9%, range 2.1–90.8%). The oral hygiene was poor (visible plaque mean 59.2%) and bleeding on probing frequent (mean 31.1%). Pathological periodontal pockets were less frequent. However, one of the participants (case 5) was diagnosed with 16 pathological pockets (out of 56 examined).Table 3 presents the salivary characteristics. The mean stimulated secretion rate was 1.3 ml/min (*SD* 0.6, median 1.1), while the mean unstimulated secretion rate was 0.08 ml/min (*SD* 0.1, median 0.06). Four participants did not produce any unstimulated saliva. The majority (*n* = 12) had a medium or high salivary buffer capacity. The total bacterial count (20^6^–320^6^) and the total number of streptococci (2.9^6^–66^6^) in the saliva were considerable. Four participants had a count of SM in the span 100,000–1,000,000 CFU/ml and six participants had ≥1,000,000 CFU/ml. Lactobacilli were present in the saliva from all participants, with half of them having a count of ≥100,000. As can be seen in Table 3, the bacterial count for most of the participants was high throughout; however, for two participants, no SM could be cultivated. Electrolyte concentrations were all within normal ranges; however, two participants scored high on fluoride content probably due to having brushed their teeth with fluoride toothpaste too close to the saliva collection.

**TABLE 2:** Dental caries, periodontal status and missing teeth

Case	Surfaces with caries (n)			Filled surfaces	Periodontal status			Missing teeth
	Early stage decay (ICDAS 1–2)	Established decay (ICDAS 3–4)	Severe decay (ICDAS 5–6)	%	Plaque index %	BOP^a^ %	Pockets^b^ n	*n*
1	1	5	4	90.8	4.1	16.7	0	4
2	12	5	1	3.3	4.1	20.8	1	3
3	24	1	0	2.1	50.0	25.0	0	0
4	24	17	7	9.2	58.3	41.1	2	0
5	12	3	0	32.1	58.3	58.9	16	0
6	1	7	2	32.2	75.0	52.8	4	10
7	17	12	4	28.4	83.3	10.0	1	8
8	6	3	3	17.7	70.8	6.3	0	12
9	4	7	3	40.9	83.3	13.0	2	5
10	2	1	4	35.0	50.0	4.0	2	3
11	8	12	0	20.0	25.0	1.8	1	0
12	15	8	9	42.5	87.5	98.1	0	2
13	9	12	9	62.9	91.6	31.0	1	7
14	44	26	10	35.6	87.5	55.6	2	1

^a^ Bleeding on probing.

^b^ Pathological periodontal pockets (≥3.5 mm).

**TABLE 3:** Salivary characteristics: Production, buffering, microbiota and electrolytes

	Flow rate			Microflora				Electrolytes				
Case	Unstimulated(ml/min)	Stimulated (ml/min)	Buffer capacity	Bacteria (CFU/ml)	Streptococci (CFU/ml)	S. mutans (CFU/ml)	Lactobacilli (CFU/ml)	Calcium (mmol/L)	Phosphate (mmol/L)	Urea (mmol/L)	Potassium (mmol/L)	Fluor (μmol/L)
1	0	0.72	Low	1.3 × 10^8^	4.4 × 10^7^	1.9 × 10^6^	9.2 × 10^6^	1.12	3.80	4.10	22.40	26.05
2	0.13	1.32	Medium	1.1 × 10^8^	3.8 × 10^7^	2.0 × 10^3^	1.4 × 10^4^	1.15	5.20	5.70	26.90	2.48
3	0.04	0.84	High	2.0 × 10^7^	2.9 × 10^6^	1.9 × 10^4^	1.3 × 10^4^	0.84	4.00	4.00	17.40	25.16
4	0	2.16	High	7.4 × 10^7^	1.4 × 10^7^	0	1.8 × 10^5^	0.91	4.20	3.10	23.20	2.47
5	0	1.50	High	9.2 × 10^7^	2.0 × 10^7^	1.6 × 10^6^	1.5 × 10^4^	0.09	4.00	2.70	25.40	1.88
6	0.09	2.56	Medium	1.2 × 10^8^	2.7 × 10^7^	2.6 × 10^5^	1.2 × 10^4^	0.97	3.50	3.80	19.90	1.72
7	0.08	1.32	High	1.8 × 10^8^	5.8 × 10^7^	2.3 × 10^6^	3.0 × 10^4^	0.92	3.10	3.90	18.20	1.56
8	0.15	1.00	Low	5.0 × 10^7^	2.5 × 10^7^	4.4 × 10^5^	9.2 × 10^4^	1.29	6.60	4.80	18.70	2.51
9	0	0.42	Medium	6.0 × 10^7^	3.6 × 10^7^	3.6 × 10^6^	6.0 × 10^5^	1.43	6.60	5.40	20.50	0.95
10	0.12	0.76	Medium	3.2 × 10^8^	4.8 × 10^7^	7.4 × 10^5^	6.4 × 10^3^	0.97	6.70	9.80	26.90	1.23
11	0.40	2.20	Medium	2.2 × 10^8^	6.6 × 10^7^	0	5.0 × 10^5^	0.87	3.90	4.30	19.60	0.85
12	0.09	0.97	Medium	9.0 × 10^7^	3.6 × 10^7^	2.0 × 10^6^	4.2 × 10^5^	0.91	3.70	9.60	22.40	0.73
13	0.04	1.00	Medium	5.8 × 10^7^	3.2 × 10^7^	3.2 × 10^5^	1.7 × 10^6^	1.00	5.00	4.10	21.90	1.85
14	0.02	1.28	High	5.8 × 10^7^	2.6 × 10^7^	1.6 × 10^6^	4.4 × 10^5^	1.14	4.10	3.90	21.30	1.06

The outcomes in patient‐reported variables are displayed in Table 4. The mean total energy intake was 1654 kcal/day (range 823–3074 kcal/day) with a mean of five meals per day. Four participants had a higher summed intake of sucrose than the Nordic nutrition council recommendation (2014), which is maximum 10% sucrose of the total energy intake.

**TABLE 4:** Patient reported outcomes, that is, dietary intake, behavior and self‐perceived oral health

Case	Diet				Oral health habits		Oral health problems			Oral health‐related quality of life
	Mean intake of kcal^a^	Meal frequency^a^ (n)	Mean disaccharide intake (g)^a^	Mean sucrose intake (g)^a^	Tooth brushing at least twice a day	Regular dental visits	Chewing difficulty	Tooth hypersensitivity daily or several times a week	Reflux	OHIP‐add
1	3074	6	141.9	115.7	Yes	Yes	Some	Yes	Yes	40
2	1443	4	42.8	29.7	Yes	No	No	No	No	8
3	1510	5	15.8	12.2	Yes	Yes	Some	Yes	Yes	24
4	^b^	^b^	^b^	^b^	No	No	No	No	No	19
5	1496	5	39.8	24.3	Yes	Yes	No	No	No	12
6	823	6	20.0	11.3	Yes	Yes	Some	No	No	28
7	1923	4	32.2	22.4	No	Yes	Some	Yes	Yes	45
8	1838	3	69.6	66.2	Yes	Yes	Some	No	Yes	34
9	1578	5	55.8	54.4	Yes	Yes	Some	No	No	7
10	1723	5	28.1	20.5	Yes	Yes	No	No	No	1
11	1257	6	21.7	16.9	Yes	Yes	No	No	No	1
12	1059	4	19.1	11.9	Yes	Yes	Some	Yes	No	44
13	1381	5	33.3	22.5	Yes	Yes	Some	Yes	No	41
14	2404	4	91.8	83.3	Yes	Yes	Some	Yes	Yes	10

^a^ Per day. ^b^Insufficient data.

A majority reported regular dental visits and tooth brushing twice daily. However, nine participants reported some difficulty chewing, and six noted problems with tooth hypersensitivity daily or several times a week. Slightly fewer (five participants) reported reflux problems but only one (participant 3) had frequent vomiting episodes (not in tables). All participants reported an impact on their OHRQoL, with a mean OHIP score of 22 with a wide range (1–44).

## 4 DISCUSSION

The overall picture from this case series was that the participants had poor oral health. They all showed manifestations of oral disease; that is, dental caries and/or periodontal disease at different stages. The salient findings were the frequent occurrence of hyposalivation and the large amounts of microbiota. The participants also perceived many impacts on their oral health. The results indicate that post‐bariatric individuals are a vulnerable group that may require special attention provided by the dental team and continuous dental care.

Many of the subjects had a lower number of retained teeth, more caries and fillings than similar age groups in the general Swedish population (Norderyd et al., 2015; Östberg, Nyholm, Gullberg, Råstam, & Lindblad, 2009). Although we did not have access to their routine dental records for verification, most reported that the reason for extraction had been dental caries. In some cases, the results may be considered contradictory, as some participants had few fillings but instead had many teeth extracted due to dental caries. Despite most participants reporting regular visits to the dentist and good oral health habits, dental plaque and gingivitis were abundant compared with the general Swedish population (Norderyd et al., 2015). The large number of restorations might have entailed challenges in dental cleaning due to crevices of the restorations.

A conspicuous finding was that most subjects (10/14) exhibited hyposalivation for unstimulated saliva (Nauntofte, Tenovuo, & Lagerlöf, 2003). To our knowledge, only one study examined unstimulated saliva in bariatric subjects finding no statistically significant differences between healthy and obese subjects, neither before nor after bariatric surgery (Knas et al., 2016). Short‐term complications after bariatric surgery comprise general dehydration (Ivanics, Nasser, Leonard‐Murali, & Genaw, 2019) which may affect salivary flow rate (Fortes, Diment, Di Felice, & Walsh, 2012). A common long‐term complication of bariatric surgery is nutritional deficiencies (Schulman & Thompson, 2017) which can affect the stimulated flow rate (Lingström & Moynihan, 2003). Also, medications used by some participants included antidepressants which may have contributed to a reduced salivary flow. The scarcity of studies of unstimulated saliva in bariatric subjects warrants further investigation. The stimulated saliva exhibited normal flow rates (Humphrey & Williamson, 2001) in line with a few other studies of bariatric subjects with short follow‐up (Farias et al., 2019). There is also a need to further follow‐up the content of substances in the saliva, such as electrolytes even if normal levels were found in the present study (Nunes, Mussavira, & Bindhu, 2015).

Another salient finding were the high bacterial counts in saliva. Especially the SM count mirrors the intake of carbohydrates and sucrose (Beighton, Adamson, & Rugg‐Gunn, 1996). The progression of the caries process is dependent of both SM and lactobacilli (Tanzer, Livingston, & Thompson, 2001) but many other bacteria may contribute in the multifactorial caries process to the onset and progression of the caries disease (Takahashi & Nyvad, 2011). This is demonstrated in case 4 and case 11: although they lacked SM in their saliva their counts of lactobacilli and total bacteria were high. Another reason for high SM counts may be that the participants may consume a more unfavorable diet than stated in the food records, where meal frequencies and calorie intake within the recommended ranges were consistent (Nordic Council of Ministers, 2014). Most methods for obtaining energy and nutrient intake are based on self‐reports, which involves limitations such as underreporting (Hill & Davies, 2001). Specifically, underreporting of food items high in fat and sugar has been found (Krebs‐Smith et al., 2000). Our analysis explored nutrient contents, not the form of food intake such as the degree of retentiveness which had been interesting as this may contribute to the risk of caries (Lingström, van Houte, & Kashket, 2000). In addition, it shall be noted that all participants were overweight or obese according to WHO definition.

The self‐reports revealed various ailments, such as tooth hypersensitivity, chewing difficulties and reflux, in line with an earlier study (Taghat, Werling, & Östberg, 2020). This was mirrored by the poor OHRQoL in accordance with a recent study showing decrease in quality of life correlated to number of perceived ailments (Gribsholt, Pedersen, Svensson, Thomsen, & Richelsen, 2016). Some of the interviewees in a Norwegian qualitative study spontaneously mentioned impaired oral health (Berg, 2020), and a Swedish survey confirmed this (Taghat et al., 2020). During the clinical visit in our study, many of the participants talked about major problems with their oral health.

The design for this study—a case series—infers no generalizable results, which is an obvious limitation of the study. Our aim was however to generate hypotheses for further longitudinal studies which can be considered achieved. Especially the frequent hyposalivation and high numbers of microbiota needs further investigation. The lack of information about the subjects' pre‐operative dental status is a disadvantage and no causation can be claimed due to the study design. The elapsed time after surgery varied among subjects which is another limitation however gained insight into possible long‐term effects of bariatric surgery on oral health.

The multidisciplinary recruitment in both general and dental health care likely provided a diversity of subjects. The uneven distribution by gender mirrors the larger number of women than men who undergo bariatric surgery (Guerra, Jean, Chiu, & Johnson, 2020; Holmberg, Santoni, Xie, & Lagergre, 2019). Selection bias may have occurred since some individuals may not have disclosed their oral status to health care professionals or, the other way around, kept information about their bariatric surgery from the dentist. This may be due to the sensitive nature of the matter. Regarding the two bariatric procedures (GBP and SG) our study reflects earlier circumstances when GBP was the predominant bariatric surgery. Whether oral problems arise after SG is largely unknown however, SG has been discussed as a greater risk for GERD (Oor, Roks, Ünlü, & Hazebroek, 2016) with subsequent possible risk for oral problems corresponding with earlier bariatric techniques f.i. gastric banding (Barbosa et al., 2009). In our study, the individual treated with SG both expressed and showed clinical signs of great oral problems. A comparison of oral health profiles in relation to surgical method was however not possible due to the uneven distribution between the two techniques. Taken together, the long‐term oral complications for bariatric patients remain to be studied.

In conclusion, individuals who had undergone GBP or SG surgery had poor clinically diagnosed oral health and perceived many oral health problems. Longitudinal studies are needed to monitor bariatric patients' oral health, from before bariatric surgery to long‐term postoperatively.

## CONFLICT OF INTEREST

The authors have no conflicts of interest to disclose.

## AUTHOR CONTRIBUTIONS

Negin Taghat: conceptualization, formal analyses, writing: original draft and final version of manuscript, funding acquisition.

Karin Mossberg: methodology—medical aspects, writing—review and editing.

Peter Lingström: methodology—dental aspects, resources, writing—review and editing.

Sofia Björkman and Anna Lehrkinder: analyses, writing—review and editing.

Malin Werling: conceptualization, writing—review and editing.

Anna‐Lena Östberg: conceptualization, methodology, data curation, formal analyses, funding acquisition, supervision, writing: original draft and final version of manuscript.

## Supporting information


**Table S1.** ICDAS coding rationale

## Data Availability

The data that support the findings of this study are available on request from the corresponding author. The data are not publicly available due to privacy or ethical restrictions.
