# Supplementary material for: Oral health profile of postbariatric surgery individuals: A case series
Source: Clin Exp Dent Res. 2021 Mar 5;7(5):811–8. doi: 10.1002/cre2.411 (PMC8543454; doi:10.1002/cre2.411)
Supplement: Supplementary file 1 — Table S1. ICDAS coding rationale [file CRE2-7-811-s001.docx]

APPENDIX

Supplementary Table 1. ICDAS coding rationale.

| Code | Visual code | Radiographic code |
| --- | --- | --- |
| 0 | Sound | No radiolucency |
| 1 | First Visual Change in Enamel (seen only after prolonged air drying or restricted to within the confines of a pit or fissure) | Radiolucency in outer 1/2 of enamel |
| 2 | Distinct Visual Change in Enamel | Radiolucency in inner 1/2 of enamel |
| 3 | Localized Enamel Breakdown (without clinical visual signs of dentinal involvement) | Radiolucency limited to the outer 1/3 of dentine |
| 4 | Underlying Dark Shadow from Dentin | Radiolucency reaching the middle 1/3 of dentine |
| 5 | Distinct Cavity with Visible Dentin | Radiolucency reaching the inner 1/3 of dentine (clinically cavitated) |
| 6 | Extensive Distinct Cavity with Visible Dentin | Radiolucency into the pulp (clinically cavitated) |
